# Desulfurization of JP-8 jet fuel: challenges and adsorptive materials

**DOI:** 10.1039/c7ra12784g

**Published:** 2018-02-14

**Authors:** Dat T. Tran, Jessica M. Palomino, Scott R. J. Oliver

**Affiliations:** Sensors and Electron Devices Directorate, RDRL-SED-E, U.S. Army Research Laboratory Adelphi MD 20783-1138 USA dat.t.tran4.civ@mail.mil +1-301-394-0273 +1-301-394-0293; Department of Chemistry and Biochemistry, University of California Santa Cruz California 95064 USA soliver@ucsc.edu +1-831-459-2935 +1-831-459-5448

## Abstract

The desulfurization of JP-8 (Jet Propellant 8) fuel is of interest to the U.S. military because of its potential use as a fuel source for solid oxide fuel cells (SOFCs). SOFCs can be used to supply a steady stream of power during military silent watch missions. Adsorptive desulfurization is a promising alternative to hydrodesulfurization, which is unable to remove refractory sulfur compounds and achieve the ultra-low sulfur levels necessary to prevent poisoning of SOFCs. Adsorptive desulfurization could be a portable, on-site process performed on JP-8 stocks already in the field. Within the vast field of fuel processing/reformation, herein we focus on the current status of adsorptive desulfurization performed on JP-8 jet fuel. Currently, the best performing sorbents are those utilizing high surface area porous frameworks with pore sizes large enough to accommodate sulfur contaminants. Additionally, a variety of metals in ionic, metallic, and oxide form serve as promising active sites within these sorbents. Most reports focus on reformation technologies and sorbent materials for gas-phase desulfurization and hydrogen purification of low-sulfur content diesel or light fraction jet fuel. JP-8 is unique to the Army in terms of supply. This review will thus focus on ongoing efforts in the room temperature liquid desulfurization of JP-8 and its higher levels of impurities that are more complex and difficult to remove.

## Abstract 2

This review describes ongoing efforts to remove the bulky organosulfur compounds from Jet Propellant 8 (JP-8) that cannot be removed by hydrodesulfurization.

## Graphical abstract

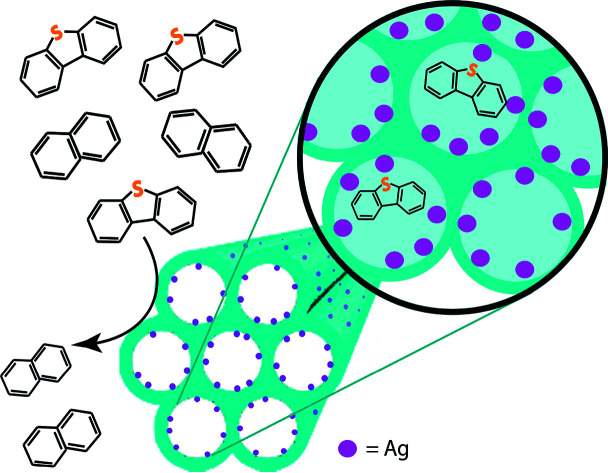

## 1. Introduction

### 1.1. Environmental and health consequences

**Scheme 1:**
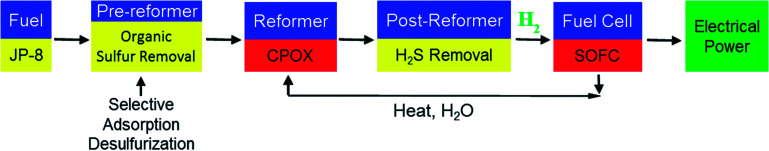
Schematic illustration of JP-8 fuel reformation for fuel cell application.

The U.S. Environmental Protection Agency (EPA) and European Union are continuously imposing more rigorous mandates for the allowable sulfur content in transportation fuel. The EPA's Tier 3 program required that gasoline will not contain more than 10 ppm sulfur by January 1, 2017, though small refiners can request up to a three year extension.^1^ Similarly, diesel fuel was capped at 15 ppm sulfur in 2006. Currently, jet fuel, including commercial and military grades, is specified to not exceed 3000 ppm_w_ S. The driving force behind the EPA's sulfur content mandates is related to health and environmental factors associated with sulfur emissions, which arise primarily from ground transportation vehicles. Burning of sulfur containing fuel causes the release of sulfur oxide (SO_*x*_) compounds into the environment. SO_2_ emissions contribute to acid rain formation, which is particularly harmful to ecosystems.^2,3^ SO_*x*_ emissions also contribute to the formation of airborne particulate matter in the atmosphere. This matter reflects some of the incoming solar radiation back into space but also poses a threat to the human cardiovascular and respiratory systems.^3–6^ It is estimated that the widespread use of ultra-low sulfur jet fuel in aviation could prevent approximately 1000 to 4000 premature mortalities annually.^7^ This short review will focus mainly on the pre-reformer that requires the adsorptive desulfurization technology (Scheme 1).

### 1.2. Fuel cell requirements

Hydrogen fuel cells provide a low emission energy alternative to traditional combustion engines, with reduced SO_*x*_, NO_*x*_, hydrocarbon and CO_2_ release. Fuel cells running on JP-8 fuel are a next generation power source for ground soldiers. In order to be easily oxidized by the fuel cell, JP-8 is converted into reformer gas (hydrogen gas and other byproducts). Impurities in the reformed JP-8 such as sulfur are poisonous to the catalysts used in the fuel cell. In addition to being a source of cleaner energy, fuel cells are also of particular interest to the military as auxiliary power units. Fuel cell power units are doubly advantageous in that they are nearly silent (compared to conventional onboard generators) and overcome the energy density limitations of conventional battery systems by utilizing the much more energy dense JP-8 fuel. These auxiliary power units would be especially useful on silent watch missions where conventional generators are too loud. Solid oxide fuel cells (SOFCs) are currently being considered by the military to fill this need.

**Fig. 1:**
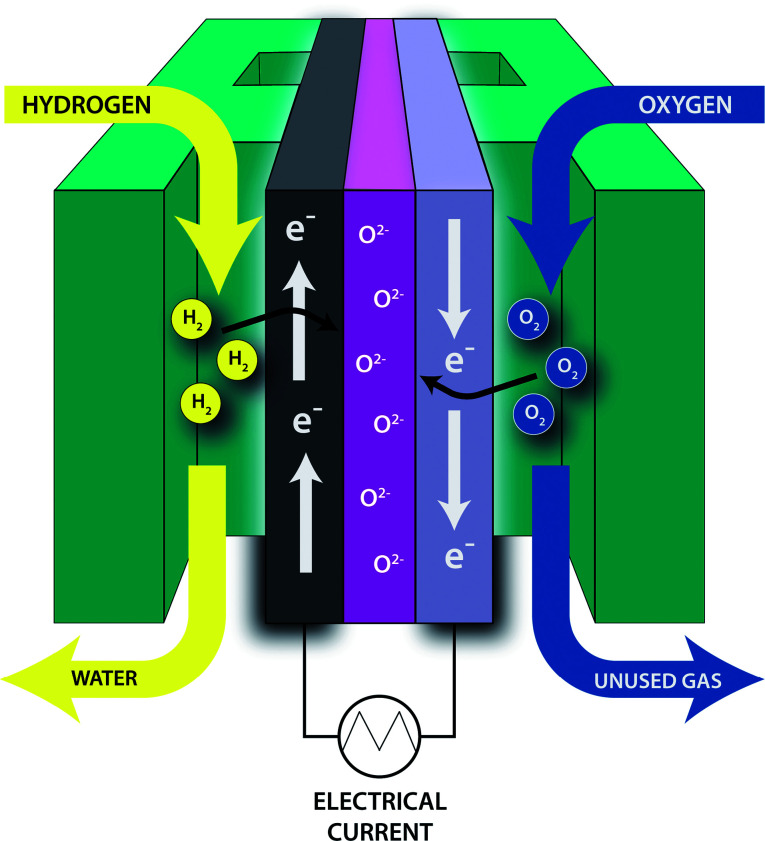
General overview of the components of a SOFC and the process for converting fuel into an electrical current. Hydrogen gas is oxidized at the anode (dark gray, left side) and oxygen is reduced at the cathode (lilac, right side). Oxygen ions are transported across the solid electrolyte (purple, center).

SOFCs are of interest because they are energy efficient, have long lifetimes and can be used with a variety of fuels.^8^Fig. 1 shows the general configuration of an SOFC. Hydrogen gas (fuel) is supplied to the anode where it is oxidized, while oxygen is supplied to the cathode where it is reduced. The anode is typically composed of nickel dispersed in a porous metal oxide cement.^8^ The cathode, which must also be porous, is typically composed of strontium-doped LaMnO_3_.^8^ A solid electrolyte, typically yttria-stabilized zirconia, allows for the oxygen ions to be transported where they can react with hydrogen ions to form water.^8^ With the addition of a catalytic partial oxidation (CPOX) reformer (Scheme 1), energy dense fuels such as JP-8 can be used as the hydrogen feedstock for SOFCs. The CPOX reformer converts the hydrocarbon chains to hydrogen gas and carbon monoxide with the addition of oxygen and a catalyst made of supported precious metals such as Rh, Pt or Pd.^9,10^ Sulfur compounds present in the fuel can poison the CPOX catalyst by binding to these precious metals, reducing its effectiveness and lifetime. Additionally, the sulfur compounds can poison the SOFC itself, binding to the nickel in the anode and decreasing the number of surface sites available for oxidation. Hu *et al.* have shown that untreated JP-8 (3000 ppm_w_ S) significantly deactivates the Pd/ZnO catalyst in as little as three hours of use.^11^ Therefore, in order to keep SOFCs efficient and cost-effective, the fuel source must be desulfurized to the lowest sulfur level as possible. SOFCs are advantageous compared to other fuel cells, not only because of their high efficiencies, but their solid construction allows them to be made in various shapes and sizes and their components are reasonably affordable. They do, however, require high operating temperatures to allow ionic conductivity, typically around 800 °C.^12,13^

The modern battlefield requires soldiers to transport energy to remote locations in the form of hydrocarbon fuels, often through dangerous territory. As energy demand rises for deployed equipment, even more energy will need to be transported in hostile environments. More efficient and energy dense fuel has the potential to reduce this heavy burden. JP-8 would be an ideal fuel source for SOFCs, as it is the main fuel source used by the military and typically already on-site for other applications. It has a high energy density and is also much safer to ship and store compared to other fossil fuels. Table 1 shows the energy density of JP-8 compared to other fuels. Its gravimetric energy density is significant compared to compressed hydrogen gas and especially lithium ion batteries. The sulfur content, however, can range from hundreds to several thousand ppm_w_ S.

**Table 1:** LHV volumetric and gravimetric energy densities of common fuel sources including JP-8 compared to that of lithium ion batteries

Fuel	Energy density (MJ L^−1^)	Energy density (MJ kg^−1^)
JP-8 (ref. 14)	34.5	43.4
Diesel^14^	36.2	42.5
Gasoline^15^	32	44
Hydrogen (liquid)^15^	120	8
Methanol^16^	15.6	19.7
Lithium ion battery^17^	N/A	0.6

### 1.3. JP-8 composition

JP-8, a military grade jet fuel, has been the main fuel used by the U.S. military for the past several decades, replacing its predecessor JP-4 as a much safer alternative.^18^ The use of JP-4 in combat situations directly resulted in causalities due to fires and explosions, prompting the necessity of a safer fuel. JP-8 has a much more favorable vapor pressure and flashpoint compared to JP-4, and is available in considerable quantities. With the exception of the Navy, JP-8 is now used in all branches of the U.S. military and on NATO bases around the world. The Navy uses JP-5 onboard its ships. JP-5 is slightly safer than JP-8, reducing the chance of fire or explosion while at sea, but is more expensive so is not used for naval ground applications or in other branches of the U.S. military.^18,19^

**Fig. 2:**
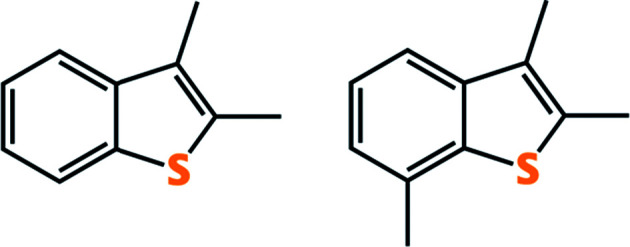
The two major refractory sulfur contaminants in JP-8: 2,3-dimethylbenzothiophene (left) and 2,3,7-trimethylbenzothiophene (right).

JP-8 is a kerosene type fuel, similar in composition to commercial Jet-A fuel but with additional additives to inhibiting and corrosion.^20^ It consists primarily of a mixture of aliphatic and aromatic hydrocarbons ranging in length from C_9_ to C_16_, including paraffins and naphthalenes.^21^ The non-sulfur components of JP-8 are highly variable, as shown by Natelson *et al.* with a sample obtained from Wright-Patterson Air Force Base and measured a pressurized flow reactor (aromatics: 16.8 vol%; olefin: 1.0 vol%; naphthalenes: 1.0 vol%; hydrogen content: 13.8 mass%; total sulfur: 0.12 mass%).^22^ JP-8 also contains a variety of organosulfur compounds, which vary based on when and where the sample was obtained. For example, Ubanyionwu and coworkers analyzed the JP-8 fuel over time from Fort Belvoir, VA.^20^ They were able to positively identify that the major sulfur contributors were 2,3-dimethylbenzothiophene and 2,3,7-trimethylbenzothiophene (Fig. 2).^20^

Materials for desulfurization are often tested on JP-5 *in lieu* of JP-8 due to availability. It should be noted that JP-5 is inherently easier to desulfurize than JP-8 due to its lower trimethylbenzothiophene content compared to JP-8. This difference is succinctly demonstrated by Tatarchuk and coworkers. An anatase TiO_2_ sorbent loaded with 4 wt% Ag had more than twice the adsorption capacity when tested with JP-5 compared to JP-8. Further, the sorbent was able to achieve sub 10 ppm_w_ S levels for JP-5 but not JP-8.^23^

### 1.4. Current desulfurization method – hydrodesulfurization

**Fig. 3:**
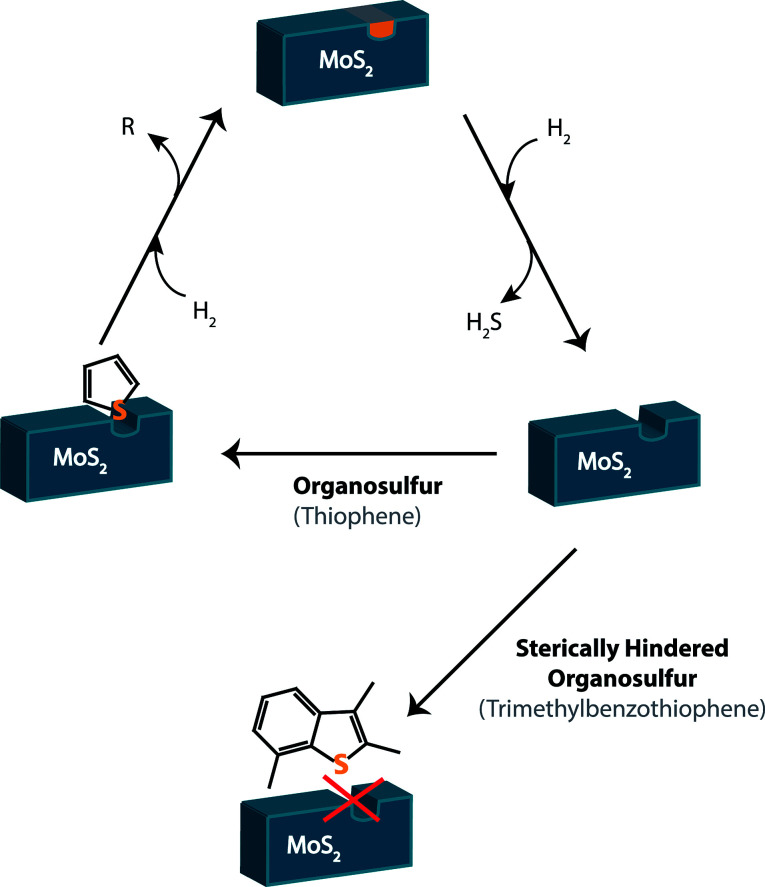
The general process for hydrodesulfurization. The MoS_2_ catalyst reacts with hydrogen to create a sulfur vacancy, where the sulfur of a non-sterically hindered organosulfur compound can bind (shown as thiophene). Upon addition of more hydrogen, hydrogenation occurs and a hydrocarbon (in the case of thiophene, butane) is released. As more hydrogen is added, the catalyst releases H_2_S again, allowing the catalyst to be reused. A sterically hindered organosulfur compound, however (shown as trimethylbenzothiophene, bottom of the figure), cannot bind to the sulfur vacancy and therefore cannot be reduced.

Currently, fossil fuels are desulfurized through the process of hydrodesulfurization (HDS). Hydrogen gas activates the catalyst – typically unsupported solid MoS_2_ – creating a coordinatively unsaturated site (Fig. 3). The sulfur atom on the organosulfur compound is able to attach to this coordinatively unsaturated site, resulting in the removal of a sulfide and hydrogenation.^24^

This process is highly effective for lighter fuels such as gasoline, where the EPA limit can easily be reached. Heavier fuels that contain larger organosulfur compounds such as methylated benzothiophenes and dibenzothiophenes, however, cannot be desulfurized by this process (Fig. 3). If the sulfur in the compound is sterically hindered, it will be unable to bind to the catalyst and thus remain in the fuel. These remaining sulfur compounds are known as refractory sulfur compounds because they remain in the fuel despite the HDS process.

**Fig. 4:**
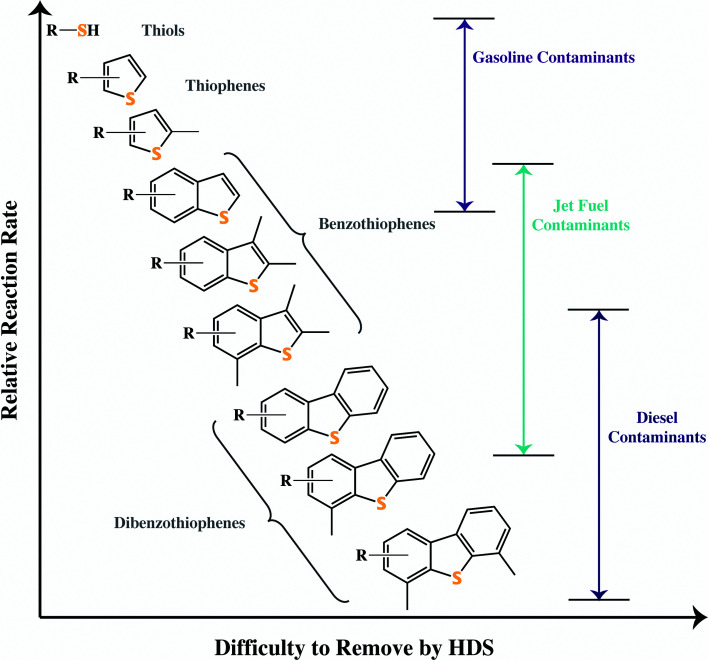
The common types of organosulfur compounds found in fossil fuels, categorized by their difficulty to be removed *via* hydrodesulfurization.

JP-8 is rich in methylated benzothiophenes and thus HDS is not an effective method of desulfurization for JP-8. Fig. 4 shows the relative effectiveness of desulfurization for a variety of organosulfur compounds found in fuels. As the organosulfur compounds become larger and more methylated, the sulfur atom becomes less likely to attach to the HDS catalyst. There are other drawbacks of HDS including non-selective hydrogenation, which for gasoline can lower the octane level of the fuel. Another is the need for hydrogen, resulting in an overall poor hydrogen economy. In addition, HDS must be performed at a refinery since it is a high pressure, high temperature process. Ideally, fuels for use in SOFCs would desulfurized onsite to specification under ambient or near-ambient conditions, as the entire stocks of JP-8 used by the military do not need to be desulfurized to the standards desirable for SOFCs (sub 1 ppm_w_ S).

## 2. Current research directions and adsorptive desulfurization frameworks for JP-8

A variety of desulfurization techniques are being explored to address the sulfur content in fossil fuels, including JP-8. Advances are being researched in hydrodesulfurization and other higher temperature catalytic processes to try and achieve lower sulfur content in JP-8,^25,26^ though at present would not be a portable process. Low temperature oxidative catalytic desulfurization processes are also being explored,^27–30^ including some involving the assistance of sonication.^31–33^ Additional exotic techniques of desulfurization have been explored on other transportation and model fuels. These less traditional techniques include extraction using ionic liquids^34–38^ and biodesulfurization using bacteria^39–42^ or enzymes.^43^ These techniques, while effective, are not as portable as adsorptive desulfurization, which would be ideal for field applications.

This review will focus on adsorptive techniques, as this process has yielded promising results and is likely the most viable for onsite applications of SOFCs in the military. Herein, we will limit our scope to techniques employed on JP-8 and light JP-8 (light refers to a lighter fraction or cut of fuel obtained by distillation), as it is the primary fuel used by the military and is more difficult to desulfurize than other military fuels such as JP-5 and commercial Jet A/B fuel. Therefore, any techniques proven useful on JP-8 will be effective on other grades of jet fuel.

There are several key qualities that are ubiquitous among desulfurization materials: (i) they tend to be porous in nature to afford very high specific surface areas, creating a significant amount of area for adsorption within a small volume; (ii) they are typically composed of low cost materials, usually metal oxides; (iii) they are usually loaded with extra-framework metal active sites for enhanced adsorption in either zerovalent, ionic or oxide form. The frameworks can have unique surface properties such as surface acidity or the ability to functionalize ligands that helps in the dispersion of the active sites or aid in the adsorption process. Lastly, these sorbents must be capable of regeneration for them to be economically viable for use by the military.

### 2.1. Nanoporous zeolites

Zeolites have been used as sorbents for a myriad of applications, as their high surface area, pore size and framework charge make them an attractive option. Their negatively charged framework allows them to easily be loaded with extra-framework transition metal ions, making them a candidate for adsorptive desulfurization.

#### 2.1.1. Zeolites for desulfurization

Zeolites loaded with a variety of intrapore, extra-framework metals have been explored for the desulfurization of fossil fuels.^44–50^ Yang and coworkers investigated Cu^+^ and Ag^+^ exchanged zeolite Y.^46,51^ The former was obtained by the reduction of Cu^2+^ exchanged zeolite Y. Cu^+^/zeolite Y can achieve sub 1 ppm_w_ S for commercial diesel (original sulfur content: 430 ppm_w_ S) and can be thermally regenerated to retain 95% of its capacity. The same material can be solvent regenerated with dimethylformamide or carbon tetrachloride, both yielding essentially 100% regeneration.^51^ Higher performance was found for Cu^+^ compared to Ag^+^ exchanged zeolites.^46,51^ Cu^+^, however, is less stable than Cu^2+^. To obtain Cu^+^/zeolite Y, Cu^2+^/zeolite Y must be reduced by heating to 450 °C in helium for 18 hours.^51^ Further zeolite testing by the same group showed that desulfurization performance follows Cu^+^ > Ni^2+^ > Zn^2+^, which agreed with their molecular orbital calculations.^44^ Cu^+^ vapor phase ion exchanged zeolite Y was explored for desulfurization of a commercial jet fuel with a similar composition to JP-8.^50^ Using fixed bed experiments, they achieved a saturated adsorption capacity of 23.2 mg S g^−1^ for commercial jet fuel, producing 38 cm^3^ of jet fuel per gram of material to an average of 0.071 ppm_w_ S (original sulfur content: 364 ppm_w_ S).^50^

#### 2.1.2. Effect of extra-framework metal on desulfurization of JP-8

**Fig. 5:**
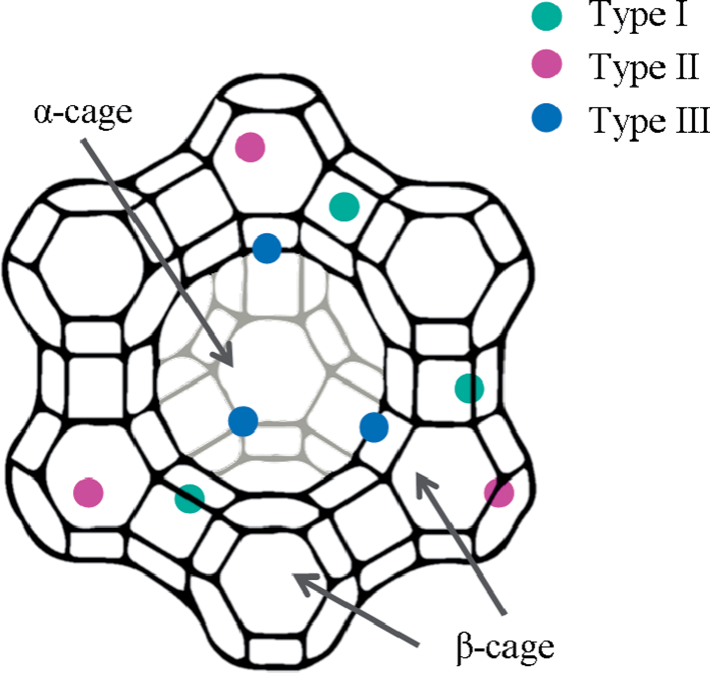
The location of different types of ion sties within zeolite Y.

Song and coworkers tested a variety of transition metal loaded zeolites with JP-8 fuel (750 ppm_w_ S) at 80 °C.^45^ Ce^3+^ and Pd^2+^ are particularly useful in the removal of organosulfur compounds, giving 2.7 and 2.6 mg S g^−1^, respectively. The Pd^2+^ exchanged zeolite, however, displays much better efficiency: it gave a nearly identical absorption capacity to Ce^3+^ despite significantly less metal loading (0.7 wt% for Pd^2+^*versus* 21.8 wt% for Ce^3+^).^45^ The authors attribute this efficiency to the possible location of Pd^2+^ in the accessible alpha supercages, whereas most of the Ce^3+^ may be in the inaccessible smaller beta cages of the zeolite (Fig. 5).^45^ They also attribute the higher efficiency of Pd^2+^ to its ability to form π-complexes, whereas Ce^3+^ likely forms only direct S–M interaction.^45^ They explored other variables such as desulfurization temperature. With the Ce^3+^ exchanged zeolite, the capacity decreased when adsorption studies were performed 40 °C and 80 °C compared to 120 °C.^45^ Overall, Pd^2+^ could make for a more affordable choice for desulfurization, even though it is more expensive than cerium: only a small amount of palladium is needed compared to cerium to achieve essentially the same adsorption capacity.

#### 2.1.3. Effect of zeolite loading procedure on desulfurization

Song and coworkers also compared two versions of Ce^3+^/zeolite Y: one calcined after exchange and one uncalcined after exchange. They found the former has a higher adsorption capacity and was attributed to higher Ce^4+^ content, which could have a more polarizing effect on the organosulfur compounds.^45^ Ion exchanged zeolite Y also performed better than zeolite Y loaded with 30 wt% Ce *via* wet impregnation. The latter they attributed to less dispersion and possibly crystallized ceria that reduces the porosity.^45^ Under the various conditions and metals explored, the lowest sulfur content obtained for 750 ppm_w_ S JP-8 was approximately 200 ppm_w_ S.^45^

Using light fractioned JP-8 and further exploring metal loaded zeolites, they were able to achieve sub ppm_w_ S fuel.^52^ The optimized adsorbent was zeolite Y that had been ion exchanged for nickel three times, followed by reduction in hydrogen at 600 °C for 4–5 hours.^52^ They again observed that ion exchange was superior to wet impregnated samples.^52^ They also found benefits of having K^+^ as a co-cation *versus* Na^+^ or H^+^. They claimed K^+^ leads to a greater number of smaller Ni particles as well as its distribution in the zeolite to prevent formation of crystal clusters.^52^ It is also thought that the metallic nickel is capable of direct S–M bonding, whereas the unreduced form leads to π-complexation for less selectivity, as there are a variety of aromatics in jet fuel.^52^

#### 2.1.4. Framework properties

Model fuel studies and molecular modeling performed with benzothiophene and dibenzothiophene on zeolite Y revealed that benzothiophene is just small enough at ∼7 Å free diameter to fit into the ∼8 Å pore free aperture of zeolite Y.^53^ While zeolite Y provides a high surface area framework for adsorption, it is likely that the larger di- and tri-methylbenzothiophenes are incapable of diffusing into the porous framework. This size exclusion would explain the above data seen by Song and coworkers. Their exchanged zeolites were only able to achieve sub ppm_w_ S levels for light fraction JP-8, which would not contain the larger, more methylated benzothiophenes. Ultimately, this size exclusion is responsible for the lower adsorption capacities of zeolites compared to the mesoporous frameworks mentioned hereafter. Overall, zeolites are an effective framework for removing small organosulfur compounds but their pore size renders them ineffective for heavier fuels such as JP-8.

### 2.2. Mesoporous silica frameworks for JP-8 desulfurization

#### 2.2.1. Mesoporous silica

**Fig. 6:**
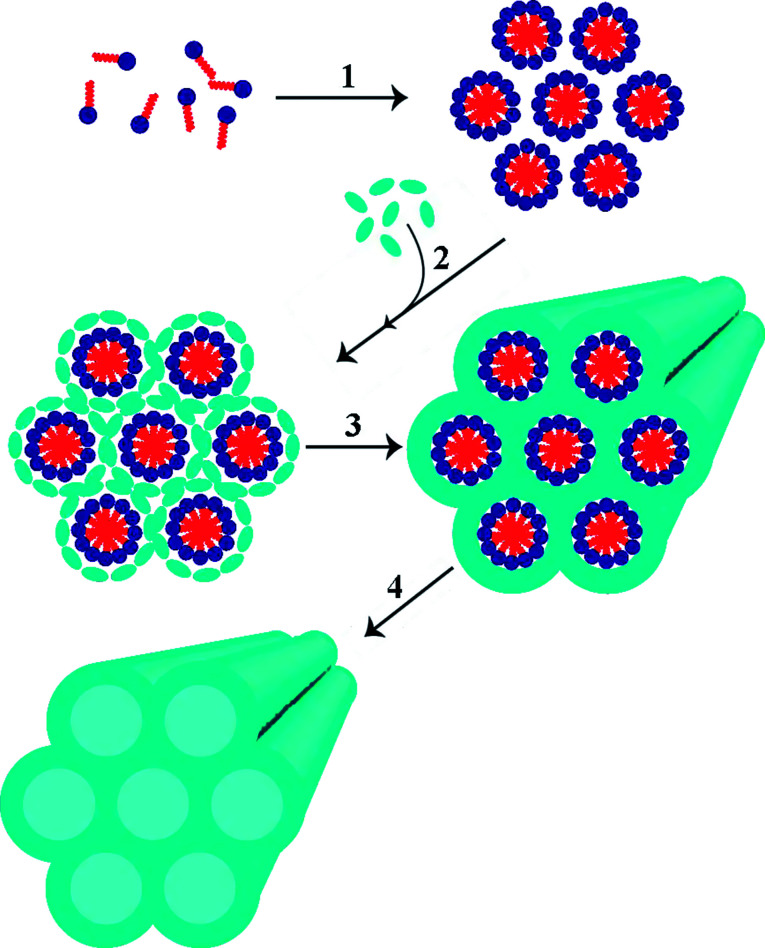
The general synthetic method for producing mesoporous silica includes the self-assembly of a surfactant (1) followed by the addition of a silica precursor (2). This precursor condenses around the surfactant (3) and the surfactant can then be removed typically *via* reflux or calcination (4) to yield the mesoporous silica framework.

Silica has been widely explored for adsorptive desulfurization in general, with a focus on mesoporous silica including MCM-41 (a hexagonal array of 1D pores, typically ∼2 to 4 nm in diameter), SBA-15 (analogous to MCM-41 with ∼4.5 to 30 nm pore diameter) and silica gel.^54–58^ Mesoporous silica is of interest because of its very high specific surface area. The general procedure for synthesizing mesoporous frameworks is shown in Fig. 6. MCM-41 or SBA-15 frameworks loaded with metal ions or metal oxides have been tested for adsorptive desulfurization with JP-5. Cu^+^ and Pd^2+^ loaded MCM-41 and SBA-15 were prepared by heating at 550 °C (helium was used for MCM-41, air for SBA-15) followed by spontaneous monolayer dispersion. This step entails mixing the metal halide and framework, then heating under helium at a high temperature for 24 hours to create a CuCl or PdCl_2_ coating.^56^ Ag^+^ and cuprous oxide loading were achieved through wet impregnation, the latter followed by autoreduction in helium.^58,59^

Pd^2+^ loaded MCM-41 was again superior to Cu^+^ for JP-5, with slightly greater breakthrough capacity (10.9 mg S g^−1^*versus* 7.7 mg S g^−1^ at 50 ppm_w_ S), saturation capacity (16.0 mg S g^−1^*versus* 14.4 mg S g^−1^) while being loaded with less metal (3.1 mmol g^−1^) than the Cu^+^ solid (5.7 mmol g^−1^).^56^ The authors attributed the better performance to the higher selectivity of Pd^2+^ because it forms stronger π-complexation than Cu^+^. Pd^2+^/SBA-15 (32.1 mg S g^−1^ at 50 ppm_w_ S and saturation capacity 38.5 mg S g^−1^) outperformed Pd^2+^/MCM-41, despite only being loaded with 16% less Pd^2+^.^56^ The higher performance is attributed to the larger pore size of SBA-15, approximately double that of MCM-41, which allows for better diffusion within the pores.^56^ The authors did not specify, however, the effect of atmosphere (air for MCM-41; helium for SBA-15) on the results. The best sorbent from the group Pd^2+^/SBA-15 was regenerated in benzene solvent at 70 °C and retained only approximately half of its saturation adsorption capacity.^56^ In contrast, Ag^+^/MCM-41 gave better results than Ag^+^/SBA-15 (breakthrough capacities 15.7 and 10.3 mg S g^−1^ at 50 ppm_w_ S and saturation capacities 32.1 and 29.2 mg S g^−1^, respectively).^58^ The authors attribute this opposite trend to the higher surface area of the loaded MCM-41 (490 and 408 m^2^ g^−1^, respectively) as well as a higher silver content (2.21 and 1.77 mmol g^−1^).^58^ Comparison of the adsorbed sulfur to silver ratios reveal similar values between the two frameworks, implying that pore size did not affect adsorption for JP-5.^58^ Ag^+^/MCM-41 was thermally regenerated in air and maintained 50% of its initial saturation capacity on both the second and third cycles.^58^

The desulfurization results of cuprous oxide supported on MCM-41 and SBA-15 confirm that the larger pore size and volume of SBA-15 is not beneficial. Rather, the higher specific surface area of MCM-41 (523 m^2^ g^−1^) makes it a relatively better adsorptive host framework than SBA-15 (400 m^2^ g^−1^).^59^ Reduction temperature was also explored, revealing that reduction at 700 °C is more effective than 550 °C, resulting in more conversion to Cu_2_O and therefore a greater adsorption capacity (12.8 and 10.3 mg S g^−1^, respectively; SBA-15 was 9.6 mg S g^−1^).^59^ Thermal regeneration was also employed on these materials, resulting in 100% regeneration.^59^

These studies reveal the superior performance of free transition metal ions compared to transition metal oxides when it comes to both saturation capacity and breakthrough capacity. SBA-15 loaded with Cu^+^ has almost three times the saturation capacity and four times the breakthrough capacity of SBA-15 loaded with almost an equivalent amount of Cu_2_O. The metal oxides are more successful, however, at regeneration. This regenerability can be attributed to the greater stability of the oxide and its stronger bonding to the surface through more covalent interactions. The discrete, free metal ions may react and be less interacting with the surface. Indeed, the process of regenerating the metal ion containing materials may produce a metal oxide. This reaction would explain the initial loss of capacity for Ag^+^/MCM-41 when thermally regenerated (conversion of Ag^+^ to Ag_2_O) and the retention of its capacity when regenerated a second time. Between the metal ions tested, Pd^2+^ is likely the best, followed by Ag^+^ and Cu^+^, although comparative testing of all three under the same conditions still needs to be studied. We also reported a mesoporous silicate templated by dodecylamine that gave an adsorption capacity of 39.4 mg S g^−1^ for JP-8 and maintained 86% of its capacity on the second cycle after regeneration with diethyl ether.^60^

#### 2.2.2. Silica gels

**Fig. 7:**
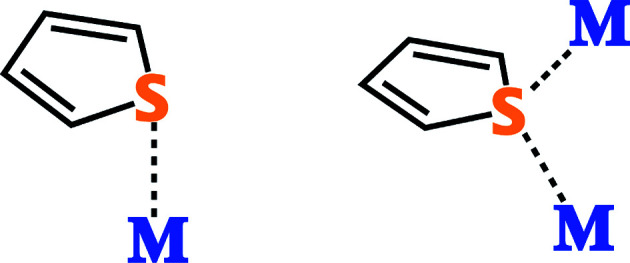
The two known coordination geometries involving direct interaction between the sulfur of a thiophene and metal center(s).

In addition to the highly ordered mesoporous silica frameworks of MCM-41 and SBA-15, silica gel is also a viable option due to its porosity, low cost and availability. Song and coworkers studied a silica gel loaded with 5.0 wt% of an undisclosed metal compound prepared by an undisclosed procedure.^61^ They report GC chromatographs of diesel fuel before and after treatment, demonstrating a reduction in the sulfur compounds present.^61^ They do not, however, quantify the reduction and simply state that similar results were obtained with JP-8.^61^ The authors performed theoretical molecular orbital calculations on thiophene, benzothiophene and dibenzothiophene. These calculations revealed that the highest occupied molecular orbital for these compounds is located mostly on the sulfur atom, suggesting that the LUMO of certain metal species interacts directly with the sulfur (Fig. 7) rather than C=C bond(s) of the ring. Direct S–M interaction is important because they can allow the selective removal of organosulfur compounds from jet fuel, which is also rich in non-sulfur hydrocarbon aromatics.^17,19^ While this preliminary study does not provide much insight into the sorbent itself, it does reveal the promise of adsorptive desulfurization as a method for obtaining ultra-low sulfur jet fuel.

#### 2.2.3. Mesoporous silica nanoparticles (MSN)

The use of nanoparticles of mesoporous silica has been very limited in the desulfurization literature until recently, despite the high surface areas afforded by these materials. Magnetic core mesoporous silica nanoparticles (Fe_3_O_4_-MSN) were reported for the removal of thiophene from isooctane.^62^ After silver loading, Ag-Fe_3_O_4_-MSN showed a modest removal of 4.7 mg S g^−1^ but exceptional regenerability, essentially maintaining its initial capacity over 6 cycles.^62^

We reported the first comparison study of commercially available bulk mesoporous silica (MCM-41) to MCM-41 nanoparticles (MSN) for JP-8 desulfurization (initial sulfur concentration 517 ppm_w_ S). Our synthesis method resulted in spherical MSN nanoparticles with an average diameter of 80 nm.^63^ Both materials were wet impregnated with a silver nitrate solution and the optimal level of silver loading was investigated with model fuel tests. Ag-MCM-41 had a maximum model fuel capacity with 18 wt% silver loading while for Ag-MSN was 20 wt%.^63^ Ag-MSN was found to have ∼30% higher adsorption capacity (32.6 mg S g^−1^*versus* 25.4 mg S g^−1^ for Ag-MCM-41) and more than a four-fold increase in breakthrough capacity (0.98 and 0.21 mg S g^−1^ at 10 ppm_w_ S, respectively). This improvement highlights the advantage of using a nanoparticle sized mesoporous material compared to a bulk powder. The adsorption capacity and breakthrough capacity of Ag-MSN are the highest reported to date for JP-8. Ag-MSN also displayed a promising regenerability of 70% on the second cycle after regeneration with diethyl ether.^63^

### 2.3. Mesoporous aluminosilicates for desulfurization

Aluminosilicates are attractive options for sorbents over silicates because they can easily be synthesized into high surface area frameworks and in the case of isomorphic substitution of Al^3+^ for Si^4+^ possess a negatively charged framework.

#### 2.3.1. Aluminosilicates *versus* silica

Along with zeolites, other aluminosilicates have been explored as adsorptive desulfurization frameworks^64,65^ and as catalytic desulfurization frameworks.^66–68^ To elucidate the desulfurization effects of the incorporation of aluminum into silica frameworks, Wang and coworkers created a series of SBA-15 materials with varying aluminum content.^65^ The SBA-15 and aluminum containing SBA-15 (which were named AS-*X*, where AS stands for aluminosilicate SBA-15 and *X* is the mass percentage of aluminum) were synthesized according to traditional methods. Pluronic P123, a triblock copolymer HO(CH_2_CH_2_O)_20_(CH_2_CH(CH_3_)O)_70_(CH_2_CH_2_O)_20_H, was the templating agent and aluminum was incorporated post synthetically using wet impregnation.^65^ Samples were then loaded with copper *via* spontaneous monolayer dispersion.^65^ Fuel tests were performed with a model fuel of thiophene in isooctane (564 ppm_w_ S). Copper loaded AS-10 gave 7.7 mg S g^−1^*versus* 5.35 mg S g^−1^ for copper loaded pure silica SBA-15, despite its decreased surface area (603 m^2^ g^−1^*versus* 816 m^2^ g^−1^ for SBA-15). This increased adsorption capacity for AS-10 was attributed to the higher dispersion of copper compared to that in the pure silica framework.^65^ There was no study on a framework-substituted aluminosilicate version of SBA-15, possibly due to difficulty in its synthesis while maintaining the large mesoporosity.

#### 2.3.2. Aluminosilicates applied to JP-8

Song and coworkers loaded a SiO_2_-Al_2_O_3_ framework with 55 wt% metallic nickel by wet impregnation followed by pre-reduction at 500 °C in hydrogen gas and passivation with hexane.^69^ The authors explored a variety of column dimensions and tested with JP-8 and light fraction JP-8 to optimize the sorbent to an adsorption capacity of 10 mg S g^−1^.^69^ For actual JP-8 they were able to get a 30 ppm_w_ S breakthrough capacity of 6 mg S g^−1^, whereas the light JP-8 had a breakthrough capacity of 16 mg S g^−1^.^69^ This paper demonstrated the possibility of fractioning before performing adsorptive desulfurization to reach lower sulfur levels.^69^ Importantly, they showed that even with unfractioned JP-8 they were able to obtain sub 30 ppm_w_ S levels, which is an important step towards the ultimate goal of sub 1 ppm_w_ S levels for SOFC applications.^69^ Also, they demonstrated the possibility of using metal(0) for desulfurization rather than metal ions or metal oxides. No regeneration, however, was reported for this material.

We recently reported a regenerable adsorbent by plasma treatment of silver impregnated mesoporous aluminosilicate nanoparticles (Ag-MASN) for the removal of dibenzothiophene from model fuel and actual JP-8.^70^ The model fuel contained an initial concentration of 500 ppm_w_ S dibenzothiopehene in decane, one of refractory organosulfur compounds in JP-8. The material is made of mesoporous aluminosilicate nanoparticles as a support where silver nanoparticles are well-dispersed and confined within the mesochannels. Our results showed an average desulfurization capacity of 15 mg S g^−1^, with 79% retention of desulfurization capacity after six cycles of dibenzothiophene removal from *n*-decane. The material was regenerated with decane rinse and plasma treatment between cycles. The material also showed a high desulfurization capacity of 41 mg S g^−1^ for actual JP-8 containing an initial concentration of 750 ppm_w_ S. The level of aluminum substitution was limited to about 2% to maintain mesoporous nanoparticles in the synthesis. It would be advantageous to find the synthetic conditions that would allow for greater aluminum doping of the host framework.

### 2.4. Functionalized silica frameworks

**Fig. 8:**
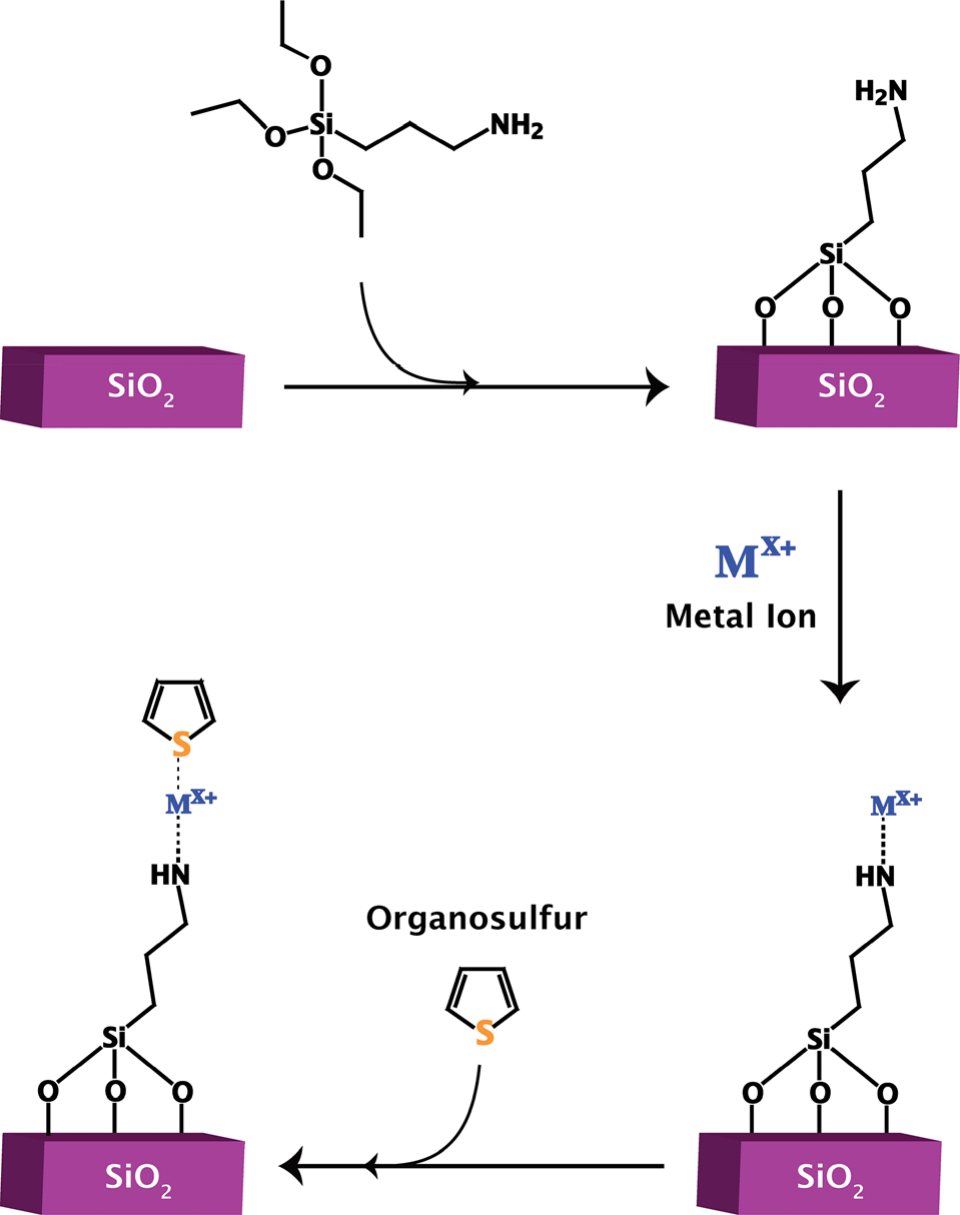
The general functionalization and metal loading of silica for sulfur adsorption.

The surface of silica is not normally very active but functionalization allows for the creation of active sites. These active sites are either those of the attached ligands themselves or those formed by attaching a binding site to the ligand, typically a thiophilic metal ion. The latter is more beneficial than loading native silica only with metal ions because it allows for better dispersion of the metal ion as well as an efficient system for anchoring the metal ion to the framework. Fig. 8 shows the general process for adding ligand functional groups to a silica surface to create a site for metal binding and ultimately an adsorptive desulfurization site.

#### 2.4.1. Functionalized silica frameworks for model fuel desulfurization

Ligand functionalized silica has been used for adsorptive desulfurization from liquid fuels,^71,72^ gas phase fuels^73^ and for oxidative desulfurization.^74–78^ Recently, Song and coworkers functionalized MCM-41 with aminopropyl groups (using 3-aminopropyltrimethoxysilane followed by Cu^2+^ loading, in a synthetic method similar to that shown in Fig. 8). The material showed promising results in the desulfurization of a light model fuel.^71^ Metal ions anchored by the ligand groups were more efficient at removing the sulfur compounds than just MCM-41 loaded with Cu^2+^. The ligand modified version removed approximately 1.8 mg S mmol^−1^ Cu whereas the ligand-free version removed approximately 0.7 mg S mmol^−1^ Cu.^71^ The authors attributed this increase to the ability of the ligand to better distribute the Cu^2+^ adsorption sites. The ligand-free version also had its copper in the form of an oxide as compared to Cu^2+^ in the ligand version, which could also account for the difference in efficiency. As seen in the previously mentioned study with MCM-41 and SBA-15, metal oxide containing materials have lower adsorption capacities than metal ion containing version.

#### 2.4.2. Functionalized silica frameworks for JP-8 desulfurization

Tran *et al.* explored ligand functionalized silica frameworks loaded with gold ions (Au^+^/SiO_2_) and compared it to silica loaded with gold nanoparticles (AuNP/SiO_2_) for the desulfurization of JP-8 (initial sulfur concentration: 430 ppm_w_ S).^72^ This metal was chosen since it can be easily deposited onto an amine-terminated siloxane monolayer first grown on the silica and allows comparison to gold nanoparticle adsorption studies. The synthesis followed the general method shown in Fig. 8. The framework was prepared by functionalizing silica gel with an average pore size of 100 Å using 3-aminopropyltriethoxysilane, followed by a 2 to 3 hour exchange with 5 mM HAuCl_4_.^72^ Gold nanoparticles were also synthesized using HAuCl_4_, sodium citrate, and sodium borohydride followed by a pH adjustment to 5 before the addition of silica. After several hours of stirring, the material was isolated, dried and calcined. Au^+^/SiO_2_ had an adsorption capacity of 5.7 mg S g^−1^ at room temperature and 6.3 mg S g^−1^ at 80 °C.^72^ Column studies revealed that Au^+^/SiO_2_ initially removes 80% of the sulfur content from the JP-8, then drops to about 70% after two milliliters have been processed.^72^ This removal represents an initial outlet sulfur concentration of approximately 80–90 ppm_w_ S. 0.3 to 0.4 g of the synthesized materials were packed into a 4.6 mm ID and 50 mm length column (Chromtech) inside a zero humidity dry room. These values are an initial example; one could always use a longer or wider column loaded with more sample. Column studies with Au^+^/SiO_2_ at room temperature and 80 °C reveal similar initial sulfur removal for the first few milliliters of processed fuel, but 80 °C extends the amount of fuel that can be processed with 70% sulfur removal. The AuNP/SiO_2_ material is saturated much more quickly. It has a similar initial removal, but quickly drops to approximately 30% sulfur removal before two milliliters have been processed. Solvent regeneration was performed using isooctane on Au^+^/SiO_2_ and demonstrated essentially 100% regeneration on the second cycle, showing no appreciable loss of adsorption.

Further studies are needed to explore the difference between gold ions and nanoparticles, as the materials did not contain the same loading content. The excellent regeneration of Au^+^/SiO_2_ is very encouraging and if the material could reach a deeper level of desulfurization, then it could be a promising adsorptive material. It is also possible that these studies could be extended to less expensive, abundant metals. Overall, the use of ligand functionalization appears to be an effective way of maintaining adsorption properties over multiple cycles, likely reducing the loss of metal during regeneration.

### 2.5. Titania

#### 2.5.1. Titania for desulfurization of JP-8

TiO_2_ (commercial available catalyst carrier, anatase phase with a surface area of 150 m^2^ g^−1^) loaded with 4 wt% Ag was found to have a saturation capacity of 2.9 mg S g^−1^ with JP-8 (initial: 630 ppm_w_ S) compared to a saturation capacity of 6.3 mg S g^−1^ for JP-5 (initial: 1172 ppm_w_ S).^23^ Breakthrough capacities for JP-5 and light JP-5 were obtained at a 10 ppm_w_ S level, whereas sub 10 ppm_w_ S concentrations were not achievable for JP-8. The latter is attributed to the trimethylated benzothiophenes that are absent in JP-5 but prevalent in JP-8. The regenerability of this material was reported only for JP-5 and breakthrough was maintained for 10 cycles *via* thermal regeneration in air.^23^ The authors decided on TiO_2_ as a framework after also investigating γ-Al_2_O_3_ and SiO_2_ as supports for silver. For a 10 ppm_w_ S breakthrough capacity, the 4 wt% Ag loaded supports ranked SiO_2_ < TiO_2_ < γ-Al_2_O_3_ and for saturation adsorption capacity ranked TiO_2_ < γ-Al_2_O_3_ < SiO_2_. These results didn't directly point to TiO_2_ as the best support but further testing showed that TiO_2_ was the best support in terms of silver dispersion and thermal stability.^23^ TiO_2_ has been recognized as a stable support for silver in previous research.^79^ Once TiO_2_ was determined to be worthwhile, the researchers further investigated the effects of surface area. Of the three TiO_2_ frameworks tested, the highest surface area material was best.^23^ Silver loading was also optimized and they found that highly dispersed silver is preferred.

#### 2.5.2. Titania surface properties for desulfurization

**Fig. 9:**
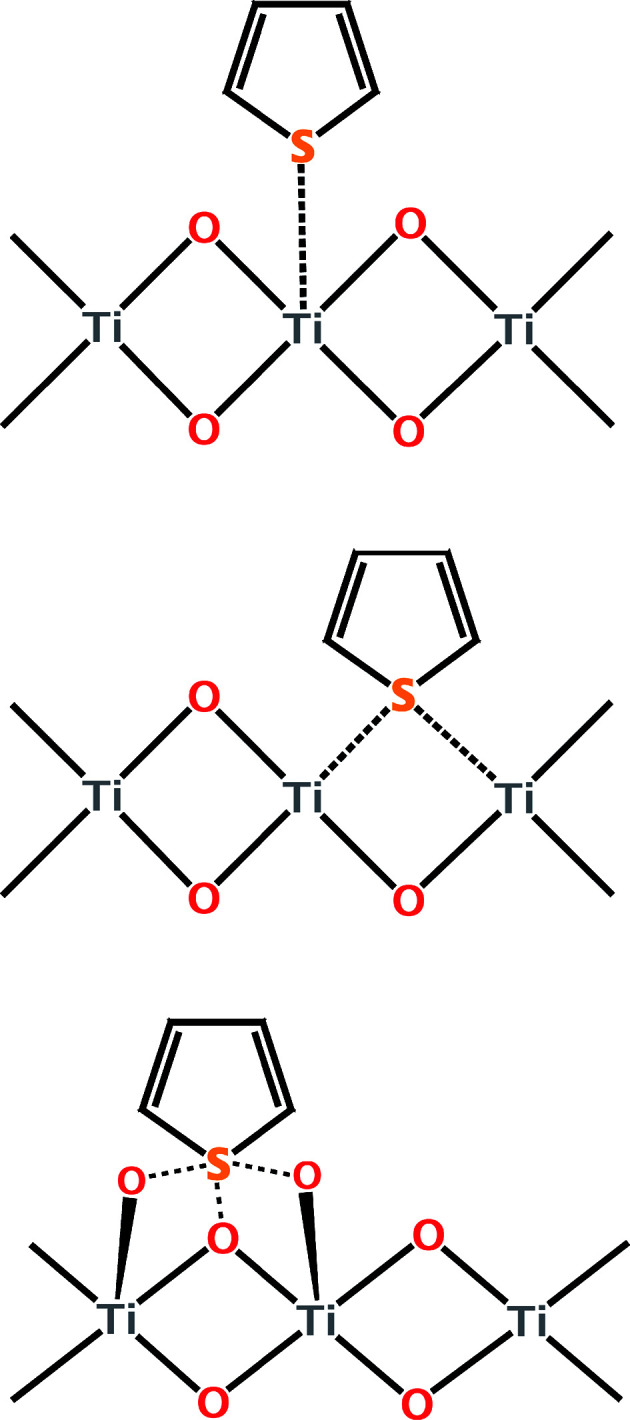
Representation of different binding configurations of thiophene on: anatase TiO_2_ (top); oxygen-poor anatase TiO_2_ (middle); oxygen-rich anatase TiO_2_ (bottom).

Titania is an attractive option for desulfurization frameworks due to its low cost and availability. It has been used primarily loaded with silver for adsorptive desulfurization.^23,80–85^ Density functional theory has been used to better understand the interactions that organosulfur compounds have with the surface of titania. Song and coworkers studied the interaction between thiophene and the (001) surface of anatase TiO_2_ (Fig. 9).^83^ They found that on perfect anatase, the strongest adsorption occurs between the sulfur and the titanium cation. For an oxygen-poor surface, the sulfur can occupy an oxygen vacancy on the surface and interact with two neighboring titaniums. For an oxygen-rich surface, they calculated that the primary interaction is between the extra oxygen atoms and no longer with the titanium. Overall, the oxygen-rich environment has the largest calculated affinity for thiophene.^83^ These findings have yet to be followed up with experimental data.

### 2.6. Titania supported on metal oxides

More commonly studied than pure titania are adsorbents made from either mixed metal titanium oxides or titania supported on other metal oxides.^86–91^ In addition to the extensive research of titania dispersed on alumina and silica that will be discussed below, titania is often mixed with ceria for desulfurization. Ceria is used because it can help lead to oxygen-rich sites that have been shown to be effective for adsorptive desulfurization (Fig. 9).^86^

#### 2.6.1. Titania supported on metal oxides for desulfurization of JP-8

Tatarchuk and coworkers prepared a variety of metal oxide supports and performed 48 hour saturation capacity experiments with JP-8.^90^ The authors tried three different titania precursors: titanium isopropoxide, titanium(iv) chloride and titanyl oxide sulfate. These precursors were loaded onto a γ-Al_2_O_3_ support *via* incipient wetness impregnation followed by calcination in air. Titanium isopropoxide produced the best results, likely due to the ease of hydrolysis of this precursor, giving a larger amount of titania dispersed on the surface. The degree of titania loading was also examined and it was found that a Ti : Al ratio of 4.4 was an optimal balance between high titania content without starting to block the pores and reduce the surface area of the γ-Al_2_O_3_ support. The optimum silver loading was between 8 and 12 wt%, as a balance of providing adsorption sites and again without starting to block pores and reduce available surface area. Thermal regeneration was performed on 12 wt% Ag/TiO_*x*_-Al_2_O_3_ and the adsorption for JP-5 remained relatively constant over 5 cycles.^90^ This result is consistent with the thermal regeneration data previously seen with other metal oxide loaded frameworks. 4 wt% Ag/TiO_*x*_-Al_2_O_3_ (Ti : Al = 1 : 4.4) had a 10 ppm_w_ S breakthrough capacity of 0.12 mg S g^−1^ with JP-8 (initial: 630 ppm_w_ S) compared to a breakthrough capacity of 0.90 mg S g^−1^ with JP-5 (initial: 1172 ppm_w_ S). The saturation adsorption capacity was 6.11 mg S g^−1^ for JP-8 compared to 10.11 mg S g^−1^ for JP-5.^90^ These adsorption capacities highlight the relative desulfurization levels between JP-5 and the more difficult to treat JP-8.

#### 2.6.2. Titania supported on metal oxides: mechanistic studies

Further investigation of TiO_*x*_-Al_2_O_3_ showed that 10 wt% is the optimal silver loading.^92^ Comparison studies using model fuels with 10 wt% Ag/TiO_*x*_-Al_2_O_3_ showed that the presence of non-sulfur containing aromatics bind to and decrease the number of available sulfur adsorption sites. In a competitive binding study between benzene and benzothiophene, benzene occupied 18% of the adsorption sites typically held by benzothiophene. A comparison between 10 wt% Ag/TiO_*x*_-Al_2_O_3_ and unloaded TiO_*x*_-Al_2_O_3_ showed that the presence of benzene decreases the capacity of the silver loaded framework, indicating the silver sites are the principle sites for π-complexation with the aromatics.^92^ IR was used to help determine the adsorption pathways. These studies showed that acidic surface hydroxyl sites were capable of initiating ring opening reactions on adsorbed thiophene and its derivatives to produce aliphatic-like compounds.^92^ Additional studies revealed that the surface acid sites interacted with the silver to aid with dispersion.^91^ In addition, the Brønsted acid sites were capable of adsorbing thiophene and create a direct interaction with the sulfur, while the silver sites (in the form of silver oxide) are capable of π-interaction with both sulfur containing and non-sulfur containing aromatics.^91^ The studies give insight into the adsorption mechanism and offer a strategy for designing improved desulfurization materials.

## 3. Army perspective of enabling desulfurization materials for future compact jet fuel (JP-8) power sources

As learned in Iraq and Afghanistan, one of the largest expenses to the Army in terms of both casualties/injuries and dollars is the transport of fuel and supplies (*i.e.* logistics convoys). Reducing the logistic burden through the integration of more efficient power sources is a primary goal of the Army. Further, future capabilities such as Silent Watch require an energy dense and silent power source.^58^ The U.S. Army has a one fuel policy, which requires the use of logistics fuel (*i.e.* JP-8) for most power and mobility applications. Our basic concept is to strip harmful impurities from the liquid fuel feed and break the complex fuel down into simpler constituents (*i.e.* reform it into hydrogen and other byproducts) that may be used by the power source. Within the U.S. Army Research Laboratory, sorbent materials that can remove organic sulfur contaminants at room temperature directly from the liquid fuel JP-8 and gas phase H_2_S at >400 °C are being developed.^93^ These materials extend the life of the reformer catalyst and enable the removal of additional fuel processing components/steps.

The problem and approach are unique to the Army. Academic institutions and industry have invested in research and development of reformation technologies and sorbent materials for gas-phase desulfurization and hydrogen purification. These organizations, however, typically deal with low-sulfur content diesel or light fraction jet fuel, which are not as complex and do not contain the impurities present in JP-8. This makes room temperature liquid desulfurization unique to the Army and the Department of Defense. To integrate advanced fuel cell power sources into the Army, advanced solutions are needed to tackle the associated problems. Within the lab, we develop sorbent materials that remove these organosulfur molecules from the fuel stream at room temperature before the reforming step and H_2_S (one of the reformate gases) after the reforming stage. These materials need to be regenerable and have a high capacity for a range of complex organosulfur compounds such that most of the contaminants may be stripped from the liquid fuel and gas phase as well.

Adsorptive desulfurization could be a portable, on-site process performed on JP-8 stocks already in the field. Currently, the best performing sorbents are those utilizing high surface area porous frameworks with pore sizes large enough to accommodate sulfur contaminants (Table 2). Additionally, a variety of metals in ionic, metallic and oxide form improve sulfur removal capacity when added to these porous frameworks by introducing more active sites. It is possible that a tandem arrangement of these materials may be necessary to achieve the continuous deep desulfurization level necessary to maintain longevity of the SOFCs. We encourage researchers to develop materials or technology in this field that will benefit the Army with the new era of power generation.

**Table 2:** Summary of the organosulfur adsorption capacities of materials towards JP-8. There have been limited studies since most reports focus on JP-5 or model fuel, which are inherently easier to desulfurize

Sorbent	Initial ppm_w_ S of JP-8	Optimized adsorption capacity (mg S g^−1^)	Reference no.
Zn^2+^/zeolite Y	750	0	45
Cu^2+^/zeolite Y	750	0.3	45
MCM-41	517	0.8	63
H^+^/zeolite Y	750	1.3	45
Ni^2+^/zeolite Y	750	2.0	45
Pd^2+^/zeolite Y	750	2.6	45
Ce^3+^/zeolite Y	750	2.7	45
MSN	517	4.6	63
Au^+^/SiO_2_	6.6	5.7	72
Ag-TiO_*x*_-Al_2_O_3_	630	8.01	92
Ag-MCM-41	517	25.4	63
Ag-MSN	517	32.6	63
DDA-15	750	39.4	60
Ag-MASN	750	41.1	70

## 4. Conclusions

The viability of adsorptive desulfurization of JP-8 to the levels required by SOFCs is promising but requires further development. Several materials have demonstrated the ability to obtain JP-8 in very low ppm_w_ S concentration but only in small quantities thus far. Some of the materials have proven themselves regenerable, in several cases even reaching 100% regenerability. Going forward, there are certain qualities that lend themselves to effective JP-8 desulfurization adsorbents. Noble metals including silver, gold and palladium seem to be the most promising active sites for future JP-8 adsorptive desulfurization and the loading must be minimized to keep cost low. Framework pore size needs to be larger than trimethyldibenzothiophene species, meaning small and mid-pore size zeolites cannot be used. MCM-41 has sufficient mesoporosity and the larger SBA-15 sized meso/macropores are unnecessary and in fact limits isomorphic substitution of aluminum and in turn metal loading. In addition, framework properties (metal oxide surface chemistry or ligand functionalization) that allow for highly dispersed metal species is a general strategy that allows for greater desulfurization capabilities. Thermal regeneration is excellent when the material is loaded with a metal oxide but this method is of lower capacity and can result in the release of sulfur compounds into the atmosphere. Solvent regeneration better isolates the organosulfur contaminants, preventing further pollution. Solvent regeneration can be used on organic functionalized materials and avoids both the oxidation of the metal ions and the need for an inert atmosphere. Another strategy that needs further study is substituting Si for aliovalent metals in the host framework. Intraframework metal may lead to greater uptake and avoid the formation of metal oxide on regeneration cycles. A combination of some or all of these strategies will likely be necessary for realizing the important goal to the Army of JP-8 desulfurization for SOFCs.

## Conflicts of interest

There are no conflicts to declare.

## Supplementary Material
